# Job Satisfaction Mediates the Association between Perceived Disability and Work Productivity in Migraine Headache Patients

**DOI:** 10.3390/ijerph16183341

**Published:** 2019-09-10

**Authors:** Isabella Berardelli, Salvatore Sarubbi, Dorian A. Lamis, Elena Rogante, Valeria Canzonetta, Andrea Negro, Martina Guglielmetti, Alice Sparagna, Valerio De Angelis, Denise Erbuto, Maurizio Pompili, Paolo Martelletti

**Affiliations:** 1 Department of Neurosciences, Mental Health and Sensory Organs, Suicide Prevention Center, Sant’Andrea Hospital, Sapienza University, 00189 Rome, Italy; 2 Department of Psychology, Sapienza University, 00185 Rome, Italy; 3 Department of Psychiatry and Behavioral Sciences, Emory University School of Medicine, Atlanta 30322, Georgia; 4 Department of Clinical and Molecular Medicine, Faculty of Medicine and Psychology, Sant’Andrea Hospital, Sapienza University, 00189 Rome, Italy

**Keywords:** migraine, work productivity, job satisfaction, perceived disability, health psychology

## Abstract

Migraine headache is the cause of an estimated 250,000,000 lost days from work or school every year and is often associated with decreased work productivity. The aim of this cross-sectional study was to assess the relationship between perceived disability, job satisfaction and work productivity in patients affected by chronic migraineurs. Participants were 98 consecutive adult outpatients admitted to the Regional Referral Headache Centre of the Sant’Andrea Hospital in Rome, Italy. Patients were administered the Italian Perceived Disability Scale, The Quality of Life Enjoyment and Satisfaction Questionnaire–Work Subscale and The Endicott Work Productivity Scale. Perceived disability is significantly associated with job satisfaction and work productivity. Job satisfaction is significantly related to work productivity and mediates the association between perceived disability and work productivity in patients affected by chronic migraineurs. Our results confirm that patients suffering from migraine headaches who have negative perceptions of their disability are less satisfied with their job, which in turn, decreases their work productivity.

## 1. Introduction

Evidence supports the notion that that 25 to 30 million people in the United States suffer from migraine headaches, and approximately 11 million of these patients have a considerable disability due to migraine attacks, resulting in a significant loss in labor costs and substantial health care expenses [1,2]. Migraine headache is the cause of an estimated 250,000,000 lost days from work or school every year [3]. According to a systematic analysis for the Global Burden of Disease Study 2016, headache disorders, and migraine in particular, are considered important causes of disability worldwide and a high-priority public-health problem [4].

Recent research has shown a complex and multifaceted relationship between psychiatric disorders and migraine, confirming high rates of comorbidity with depressive and anxiety symptoms [5,6,7], which inversely could contribute to headache progression and chronicity [8]. Attempted suicides also seem to be increased in patients suffering from migraine compared to the general population [9,10]. 

Migraine is a cyclical illness that needs correct treatment of acute attacks, but also adequate treatment prophylaxis to reduce the intercritical pain [11]. Bodily pain, nausea, vomiting, and other symptoms that occur during migraine attacks result in impairments in both job and social functioning, considerably reducing the quality of life of patients [12]. Studies show that the frequency of migraine attacks is inversely related both to quality-of-life and disability, and a positive relationship between disability and emotional distress in chronic daily headache patients has been well documented [13,14]. Moreover, Huang et al. [15] showed that individuals with chronic migraine had significantly lower scores in a screening instrument that assesses general cognitive abilities, specifically in language, executive functions, calculation, memory, and orientation domains.

According to social cognitive theory, the use of positive psychological coping strategies influences the perceptions of self-efficacy of patients. Moreover, the concept of self-efficacy is closely linked to perceived disability and influences an individual’s adaptation to a medical disease by impacting cognitive, affective, physiological responses, as well as the initiation and persistence of efforts to prevent headache episode. French et al. [16] assessed the link between self-efficacy and perceived disability in the prevention and management of headache episodes. In this context, a high perceived disability, defined as patient’s beliefs about the illness, could strengthen the impact of migraine on daily life activities, work productivity, and appropriate treatment [17].

Job satisfaction has been proposed to be a multi-dimensional concept of employees’ feelings related to work and the working environment [18]. The level of job satisfaction can affect the health and well-being of employees and can also influence an employee’s productivity [19]. Data on the relationship between mental health and job satisfaction has revealed that job satisfaction was lower among employees with severe psychological distress and is significantly related to psychosomatic problems [20,21,22].

Evidence suggests that migraine is associated with job satisfaction and worker productivity [23]. Furthermore, research has demonstrated that effective therapy for migraine can reduce loss in worker productivity [24]. 

Previous trials reported a significative association between job dissatisfaction, mental health, social action, and depression in workers [22]. However, the level of job satisfaction should be a key factor influencing the health of workers [19]. Stewart et al. [25] reported mean productivity losses of 4.7 hours/week for migraine in the United States. In an observational study [26] among a young mostly male workforce, results confirmed findings from a previous trial demonstrating a significant decrease in working productivity due to headache [27,28]; the authors revealed that the decrease of productivity was higher in employees who experienced a higher frequency of headaches.

Given the fact that migraine is often associated with decreased work productivity, this study was designed to assess the relationship between perceived disability, job satisfaction, and work productivity in patients affected by migraine. We hypothesized that: (1) Perceived disability would be significantly associated with job satisfaction and work productivity; (2) Job satisfaction would be significantly related to work productivity; and (3) job satisfaction would significantly mediate the association between perceived disability and work productivity.

## 2. Materials and Methods 

### 2.1. Participants

Participants were 98 consecutive adult outpatients (85 women, 13 men) admitted to the Regional Referral Headache Centre of Sant’Andrea Hospital in Rome, between May 2017 and September 2017. All participants were diagnosed with chronic migraine according to diagnostic criteria of ICHD 3β [29] and were in treatment with 195 U of OnabotulinumtoxinA. The average age of participants was 48.33 years (*SD* = 9.87). Fifteen patients (15.3%) had a secondary school education, 52 patients (53.1%) had a high school education, and 31 patients (31.6%) reported obtaining a college degree. Seventy-six (77.6%) participants were married, whereas 22 (22.4%) were not married (see Table 1). None of the patients had significant medical problems other than migraine or stress-related disorders.

Health care staff have sensitized patients about the relevance of the study, explaining goals and purposes to ease aware subscription to the research. 

Inclusion criteria: All patients with chronic migraine undergoing treatment with OnabotulinumtoxinA, with the ability to carry on a conversation, cognitive skills to fully understand the instructions and the questionnaires, and they were older than 18 years

Exclusion criteria were the presence of a Montreal Cognitive Assessment (MoCA) score lower than 24 or a Frontal Assessment Battery (FAB) score lower than 12.

The subjects were enrolled while they were waiting to undergo the treatment with botulinum toxin. A formed psychologist has acquired consent forms and carried out the interviews. Each participant was informed about the aims and purposes of the study, and the anonymity was assured assigning an alphanumeric code. All the patients gave their written informed consent before participating. The study was approved by the Board of Ethical Committee (Prot. n. 33 SA_2019).

### 2.2. Measures

The **Italian Perceived Disability Scale** (**IDPS**) [30] is a 20-item self-report instrument assessing headache-related disability on a 5-point Likert scale (*completely false* to *completely true*). Specifically, the IDPS consists of items inquiring about people’s beliefs regarding autonomy/disability in different situations of life. Sample items include “my body is weak and unreliable” and “I will have to worry about my health conditions all my life long.” The IPDS is easy to administer and useful for assessing disability in chronic daily headache patients. The IPDS has been shown to have strong psychometric properties, including solid validity and reliability [30]. In the present study, the reliability estimate was 0.94.

The **Quality of Life Enjoyment and Satisfaction Questionnaire—Work Subscale** (**Q-LES-Q**) [31] is a 12-item self-report measure of the degree of enjoyment and satisfaction experienced in one’s job/work. Items are rated on a 5-point scale from 1 (*not at all or never*) to 5 (*frequently or all of the time*), with higher scores denoting greater levels of job satisfaction. An Italian version of the Q-LES-Q, which was administered, has been shown to be a valid and reliable measure [32] and has been used effectively in headache patients [33]. In the present study, the Cronbach alpha was 0.91.

The **Endicott Work Productivity Scale** (**EWPS**) [34] was developed to quantify the frequency of work performance and productivity attitudes and behaviors over a 1-week period, for a broad range of diseases and occupations [35]. The 25-item EWPS assesses four domains, including attendance, quality of work, performance capacity, and personal factors (social, mental, physical, and emotional). Response options range from 0 (*never*) to 4 (*almost always*), and the scale score is calculated out of 100, with 100 representing lowest productivity [34]. Sample items on the EWPS include “How frequently do you just do no work at times when you would be expected to be working?” and “How frequently do you work more slowly or take longer to complete tasks than expected?” Previous studies have shown that the EWPS has strong psychometric properties, including validity and reliability [36,37]. In the present study, the internal consistency reliability estimate was 0.86.

### 2.3. Statistical Analyses

Pearson’s correlations were calculated to examine zero-order correlations between study variables. The key hypotheses were evaluated in a single, saturated (i.e., just-identified) path analytic model, with age, gender, and education level modeled as exogenous covariates predicting all study variables. Model fit indices are not presented due to the just-identified nature of the model. Although a full structural equation model (SEM) would have minimized measurement error, the current sample size was not deemed large enough to estimate a measurement model effectively [38]. Mediated paths and total effects were tested as the product of coefficients in a single saturated path model estimated in Mplus v.7.4 [39], using the Full Information Maximum Likelihood (FIML) estimation feature in Mplus to accommodate for any missing data. The null hypothesis is that the sum of the two indirect paths—from the predictor (perceived disability) to the mediator (job satisfaction) and from the mediator to the outcome (work productivity)—is equal to zero, indicating no indirect effect. 

As described in any standard treatment of indirect effects [38,39,40], the model was a conventional three-variable mediation system, with the addition of the suite of covariates. We tested for the significance of indirect (mediated) effects using the percentile bootstrap with 3000 draws to generate empirical confidence intervals for the products of the coefficients composing the mediated paths. Compared to casual step approaches to mediation, such as bootstrapping, a nonparametric resampling technique has been shown to be more robust against normality violations, yield higher estimates of statistical power, and demonstrate greater control over Type I error rates [38]. Moreover, bootstrapping procedures are less sensitive to specification errors [41,42]. Overall, when compared to more traditional approaches of assessing mediation, the bootstrapping procedure has been shown to be a statistically appropriate method and strongly recommended for testing indirect effects [2].

## 3. Results

**Figure 1:**
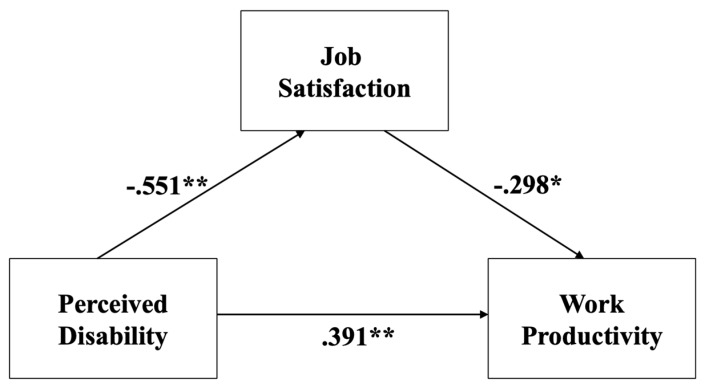
The model with standardized regression coefficients depicting job satisfaction as a mediator in the relation between perceived disability and work productivity. *Note.* The analysisincludes age, sex, and education level as covariates modeled as exogenous variables. * *p* < 0.05; ** *p* < 0.01.

Descriptive statistics and two-tailed correlations among the primary study variables— perceived disability, job satisfaction, and work productivity—are presented in Table 2. All bivariate and partial correlations were significant at *p* < 0.05 in the expected direction. Although these results support Hypothesis 1 and 2, we further tested the predictive relations among study constructs in the context of the mediational model while adjusting for relevant covariates. The model is diagrammed in Figure 1, with standardized coefficients shown. In the mediational model and consistent with our hypotheses, the path coefficient between perceived disability and job satisfaction was significant (*b* = −0.239, 95% CI [−0.35, −0.12]); the path coefficient between perceived disability and work productivity also was significant (*b* = 0.265, 95% CI [0.07, 0.46]); and the path coefficient between job satisfaction and work productivity was significant (*b* = −0.464, 95% CI [−0.91, −0.05]).

The primary hypothesis (Hypothesis 3) focused on the mediation of the link from perceived disability to work productivity through job satisfaction. In the model, the total effect of perceived disability on work productivity was positive and significant, with a point estimate of 0.376, 95% CI [0.21, 0.54], and standardized estimate of 0.56. Consistent with Hypothesis 3, this effect was significantly mediated by job satisfaction, *ab* = 0.11, 95% CI [0.01, 0.25], which revealed a medium effect size for the indirect effect [43,44]. The confidence interval excluded zero, indicating a significant indirect effect of perceived disability on work productivity via job satisfaction, supporting the mediation hypothesis. Moreover, the standardized effect size for the indirect effect was 0.16, indicating that work productivity decreases by 0.16 standard deviations for every 1-SD increase in perceived disability indirectly via reduced job satisfaction, after accounting for several important covariates. In other words, patients suffering from migraine headaches who have negative perceptions of their disability are less satisfied with their job, which, in turn, decreases their work productivity. 

## 4. Discussion

In this study, we investigated the association between perceived disability, job satisfaction and work productivity in patients affected by a migraine headache. Moreover, we examined job satisfaction as a potential mediator in the relationship between perceived disability and work productivity. Clinical studies have reported conflicting results regarding the relationship between frequency and intensity of the headache, psychiatric comorbidity, and disability [45,46]. For example, in a recent trial of women affected by episodic migraine, the researchers did not find any significant associations among pain extent, migraine pain features, or psychological variables including anxiety or depression and migraine-related-disability [47].

According to the work of Locke and colleagues [48], the concept of job satisfaction should be considered as an emotional situation resulting from job experiences, evaluation of one’s work, personal appraisal of job, and career experiences. Although job satisfaction was associated with reduced psychological distress [49], no studies have investigated job satisfaction, physical health symptoms, and perceived disability. Few studies have investigated job satisfaction in patients affected by medical diseases [50,51], which examined the effects of potentially traumatic events, post-traumatic stress symptoms, and coping self-efficacy on job satisfaction. The results from these studies demonstrated that only job satisfaction before the traumatic event was a predictive factor for job satisfaction after a traumatic event [51]. Fiabane et al. [50] explored predictors of job satisfaction among cardiac patients who have returned to work after cardiac rehabilitation; baseline job satisfaction, depression, and ambition turned out to be significant predictors of job satisfaction following return to work. When job satisfaction decreased, phenomena such as work-related stress and fatigue increased [28]. A recent study found that the relationship between personal experience and job satisfaction was mediated by life satisfaction in patients with disabilities [52]. Recent studies in other populations of patients affected by organic diseases identified factors associated with work disability; however, all of these studies only consider the presence of depressive symptoms and fatigue and no other psychological features [53,54].

In our study, we have therefore confirmed data in the extant literature concerning perceived disability, job satisfaction, and work productivity in patients affected by headache. Specifically, our results revealed that these three factors are connected to each other while highlighting the importance of job satisfaction as a key factor involved in the genesis of perceived disability. However, given the social and working impact of this disease, further controlled studies are needed to either replicate or refute the present findings 

This study is not without limitations. First, a comparison population is missing; patients with another disabling organic disease and a healthy control population would have been valuable. Second, this is a cross-sectional study and, by definition, cross-sectional studies have no dimension of time, so they cannot support conclusions on the risk of disease, nor on causal relationships. Third, there was not a psychiatric assessment using standardized tools, and we did not assess depressive symptoms. Fourth, we did not use measures such as the MIDAS (Migraine Disability Assessment), specifically devised for migraine and universally accepted as an instrument for assessing disability in this population. However, our choice to assess perceived disability with a general measure allows us to compare chronic migraine with other medical conditions to assess differences. We used only self-report measures which are potentially affected by social desirability bias. For the purpose of the paper, we used three standardized scales, but job satisfaction may be influenced by other factors, including personal life events and other professional influences, not tested by the scales we used. Finally, to limit the variability of the neurological condition, we tested only patients with a diagnosis of chronic migraine, and the assessment was performed when botulinum toxin therapy produced an amelioration of the symptoms. 

Investigating clinical and social features predicting perceived scale disability in patients affected by migraine headache may contribute to reducing job dissatisfaction and consequently loss in worker productivity. Job satisfaction was considered a mediator in the relationship between the perception of a discriminatory work and employees’ health [55]. Therefore, it is important to identify possible antecedent features which should have a positive effect on employees’ job satisfaction with the purpose of preventing employment discrimination, even because the perception of discrimination can become a risk factor for mobbing and burnout syndrome.

Furthermore, a better definition of the various aspects of psychiatric comorbidity that can affect the perceived disability of patients is important for reducing both the chronicity and the disability of this disease. Authors should discuss the results and how they can be interpreted in perspective of previous studies and of the working hypotheses. The findings and their implications should be discussed in the broadest context possible. Future research directions may also be highlighted.

## 5. Conclusions

Our study shows that the level of job satisfaction influences the impact of perceived disability on work productivity. It seems that individuals perceiving their migraine as more burdensome have lower levels of gratification derived by their job, which in turn has a negative effect on work productivity. 

## Figures and Tables

**Table 1:** Socio-demographical and clinical data.

Socio-Demographical and Clinical Data	Patients (*n* = 98)
Age (years)	48.33 ± 9.87
Sex	
Male	13 (13.3%)
Marital Status	
Single	11 (11.2%)
Married	76 (77.6%)
Divorced	10 (10.2%)
Living Situation	
With partner	59 (60.2%)
With family	30 (30.6%)
With friends	1 (1%)
Alone	7 (7.1%)
Education	
Secondary School	15 (15.3%)
High School	52 (53.1%)
Degree	31 (21.6%)
Days of migraine for a month	8.04 ± 5.67 (1–29)
Age of onset	17.81 ± 10.65 (5–56)

**Table 2:** Correlation Matrix, Means, and Standard Deviations of Study Measures.

Variable	1	2	3
Perceived Disability	--		
Job Satisfaction	−0.52 **	--	
Work Productivity	0.54 **	−0.48 **	--
Mean	28.88	49.31	17.01
SD	17.96	7.79	12.15

Tabled values are zero-order correlations. * *p* < 0.05; ** *p* < 0.01.
